# Comprehensive review of the traditional uses and the potential benefits of epimedium folium

**DOI:** 10.3389/fphar.2024.1415265

**Published:** 2024-09-11

**Authors:** Jialu Cui, Lin Lin, Feiran Hao, Zhuo Shi, Yehui Gao, Tingyu Yang, Chunqi Yang, Xiangjun Wu, Rong Gao, Yi Ru, Fangyang Li, Chengrong Xiao, Yue Gao, Yuguang Wang

**Affiliations:** ^1^ Institute of Traditional Chinese Medicine, Tianjin University of Traditional Chinese Medicine, Tianjin, China; ^2^ Department of Pharmaceutical Sciences, Beijing Institute of Radiation Medicine, Beijing, China; ^3^ School of Pharmacy, Henan University, Kaifeng, China; ^4^ School of Traditional Chinese Medicine, Guangdong Pharmaceutical University, Guangzhou, China

**Keywords:** epimedium folium, strengthening yang, anti-osteoporosis, anti-tumour, anti-inflammation, anti-virus

## Abstract

Epimedium Folium has been extensively utilized for medicinal purposes in China for a significant period. This review undertakes a comprehensive examination of literature pertaining to Epimedium and its metabolites over the past decade, drawing from databases such as PubMed. Through meticulous organization and synthesis of pertinent research findings, including disease models, pharmacological effects, and related aspects, this narrative review sheds light on the principal pharmacological activities and associated mechanisms of Epimedium in safeguarding the reproductive system, promoting bone health, mitigating inflammation, and combating tumors and viral infections. Consequently, this review contributes to a more profound comprehension of the recent advances in Epimedium research.

## 1 Introduction

The botanical drug Epimedium Folium, known by various names such as “Xian Lingpi” and “nine leaves on three stems,” is derived from the dried leaves of *Epimedium brevicornu Maxim., Epimedium sagittatum* (Sieb. et Zucc.) Maxim., *Epimedium pubescens* Maxim. or *Epimedium Koreanum* Nakai. This traditional tonic Chinese medicine was first documented in the Shennong Materia Medica Classic and is widely distributed throughout China. It thrives in temperate and subtropical regions, exhibiting a preference for shaded and humid environments. Epimedium Folium typically grows in the understory of forests, in shrubs along ditches, or in damp areas on mountain slopes.

**FIGURE 1:**
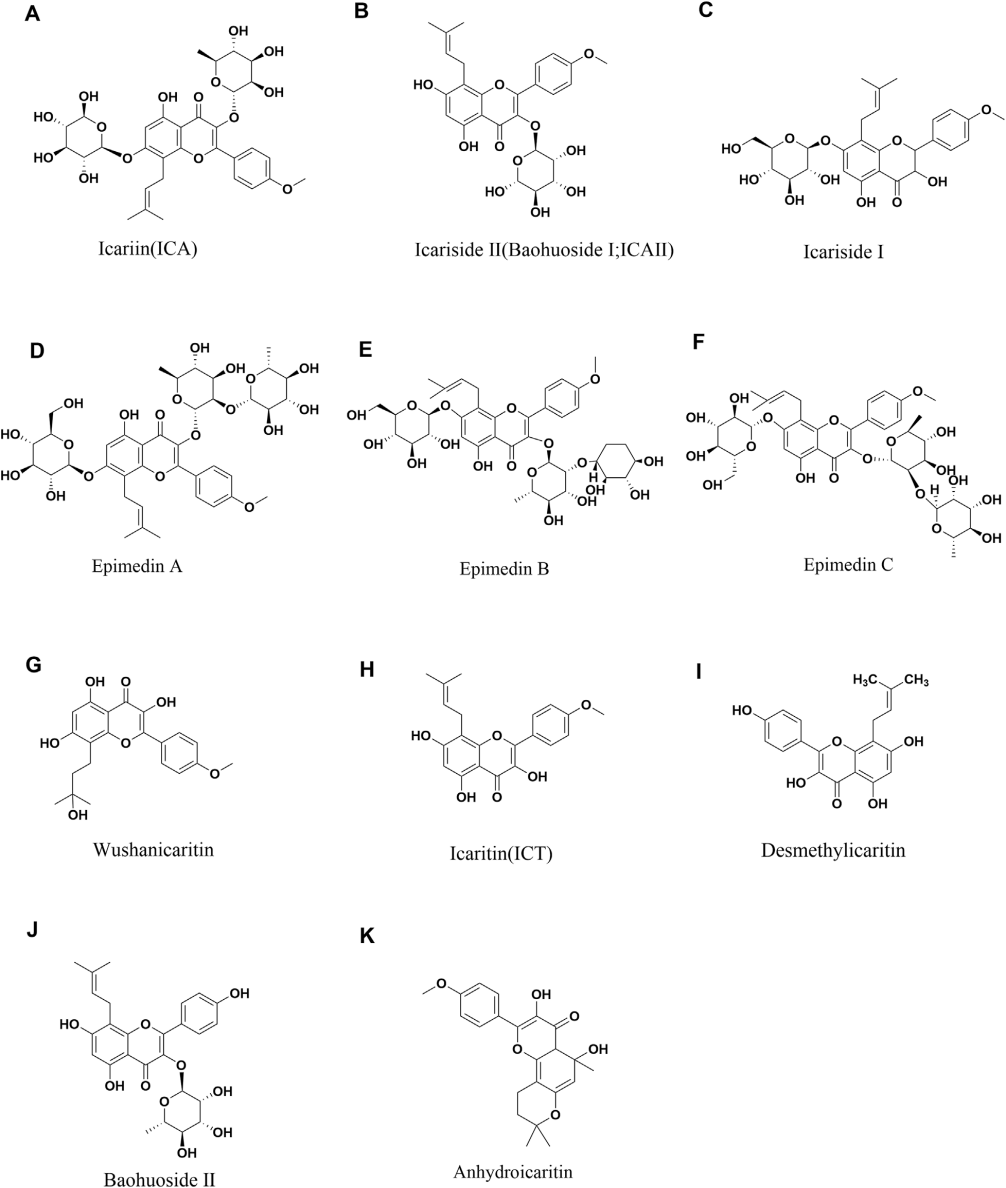
Chemical structures of Icariin **(A)**, Icariside II **(B)**, Icariside I **(C)**, Epimedin A **(D)**, Epimedin B **(E)**, Epimedin C **(F)**, Wushanicaritin **(G)**, Icaritin **(H)**, Desmethylicaritin **(I)**, Baohuoside II **(J)**, Anhydroicaritin **(K)**.

Epimedium Folium is frequently employed in China, Japan, and South Korea for its medicinal properties, including kidney tonification, aphrodisiac effects, muscle and bone strengthening, as well as wind and dampness dispelling. Contemporary research on Epimedium has led to the isolation of flavonoids, lignans, and alkaloids from its aerial parts. More than 130 different plant metabolites have been identified from Epimedium Folium, including flavonoids, icarisides, and other types of compounds (Wu et al., 2003). Among these plant metabolites, flavonoids exhibit high pharmacological activity. Icariin, Epimedin A, Epimedin B, Epimedin C, Icariside II (Baohuoside II), and Icaritin are all flavonoids found in Epimedium and are also its main pharmacologically active metabolites (Chen et al., 2015). Icariin, Epimedin A, Epimedin B and Epimedin C are used as quality control markers for Epimedium, according to the Chinese Pharmacopoeia (2020 Edition) (The Free Encyclopedia, 2020) (Figure 1) (Table 1). In addition, polysaccharides are also active substances in Epimedium. Epimedium polysaccharide is a complex carbohydrate mainly composed of mannose, rhamnose, galacturonic acid, glucose, and galactose (Ke et al., 2023). It has various pharmacological activities such as regulating immunity (He et al., 2020) and antioxidant effects (He et al., 2020).

**TABLE 1:** The important metabolites isolated from the genus Epimedium.

Compounds	Chemical substances	Species	References
Icariin (ICA)	Flavonoids	*Epimedium koreanum*	Pei-Shan et al. (2010)
		*Epimedium sagittatum*	Yuru et al. (2024)
Icariside II (Baohuoside I)	Flavonoids	*Epimedium koreanum*	Xiang Dong et al. (2017)
		*Epimedium sagittatum*	Zhu et al. (1993)
Icariside I	Flavonoids	*Epimedium koreanum*	Li et al. (1995a)
		*Epimedium sagittatum*	Mizuno et al. (1987)
Epimedin A	Flavonoids	*Epimedium koreanum*	Oshima et al. (1987)
		*Epimedium sagittatum*	Wang and Geng (2005)
Epimedin B	Flavonoids	*Epimedium koreanum*	Oshima et al. (1987)
		*Epimedium sagittatum*	Wang and Geng (2005)
Epimedin C	Flavonoids	*Epimedium koreanum*	Oshima et al. (1987)
		*Epimedium sagittatum*	Wang and Geng (2005)
Desmethylicaritin	Flavonoids	*Epimedium koreanum*	Li et al. (1996c)
Icaritin	Flavonoids	*Epimedium koreanum*	Li et al. (1995a)
BaohuosideII (Ikarisoside A)	Flavonoids	*Epimedium koreanum*	Xu and Yang (2005)
Wushanicariin	Flavonoids	*Epimedium breviconu*	Liang et al. (1988), Yan et al. (1998)

A range of *in vivo* and *in vitro* experiments, along with clinical applications, have substantiated the reproductive, osteoprotective, anti-tumour, anti-inflammatory, and anti-oxidative stress biological activities exhibited by Epimedium Folium. For example, it achieves bone protection by maintaining bone metabolism balance (Xu et al., 2012) and other pathways, reproductive protection by inhibiting PDE5 activity (Chen et al., 2009) or repairing penile tissue, and anti-tumor and anti-inflammatory effects by regulating pathways such as PI3/AKT and oxidative stress. This paper will review the pharmacological activities and related mechanisms of Epimedium Folium.

## 2 Traditional uses

In the theory of medicinal properties of traditional Chinese medicine, it is believed that the taste of *Epimedium Folium* is spicy and sweet, and its properties are mild. During the Northern and Southern Dynasties, there was a legend that a shepherd discovered that after consuming a wild grass growing in bushes, the number of penile erections and mating with ewes increased in male sheep. The shepherd told this phenomenon to the famous medical expert *Tao Hongjing*. After repeated observation and verification by *Tao Hongjing*, it was found that this wild grass did indeed have an aphrodisiac effect. Therefore, the wild grass was named “*Epimedium Folium*” and recorded in the pharmacopoeia. In addition, the famous Tang and Song poet *Liu Zongyuan* also used Epimedium to treat leg diseases caused by rheumatism. So *Epimedium Folium* is known for its effects of tonifying the kidney and strengthening yang, as well as dispelling wind and dampness, and so on. In the traditional use of *Epimedium Folium*, it can be used in combination with kidney-tonifying and aphrodisiac botanical drugs such as *Rehmanniae Radix Praeparata*, *Lycii Fructus*, and *Curculigo orchioides* (Ma et al., 2011a). Additionally, it can be made into Epimedium Folium wine, as recorded in the *Food Medicine Heart Mirror*, by soaking *0.5 kg* of *Epimedium Folium* in *5 kg* of alcohol for 10 days and consuming it daily to treat bone pain, numbness, and impotence.

*Epimedium Folium* has garnered significant attention from researchers for its advantages in treating bone and reproductive diseases, leading to extensive scientific research on its effects. *Epimedium Folium* has the potential to reduce bone loss, increase bone density, and act as a natural PDE5Is. *Epimedium Folium* and its plant metabolites maintain the balance of human bone metabolism and regulates the differentiation of BMSCs, exerts bone protective effects and shows therapeutic effects on ED by regulating the secretion of hormones involved in male erections and cell growth. Furthermore, ongoing research has revealed that *Epimedium Folium* and its plant metabolites possess significant anti-tumor (Sun et al., 2015), anti-inflammatory (Huang et al., 2018), and antiviral (Cho and Ma, 2022) activities.

## 3 Pharmacological activity

### 3.1 Bone protection

A healthy bone is dynamically active tissue, and its activity largely depends on the homeostatic bone metabolism in the body, which is the dynamic balance between the functions of osteoblasts and osteoclasts. Epimedium exerts osteoprotective effects on bone metabolism in multiple ways.

#### 3.1.1 Bone protection based on osteoporosis models

Osteoporosis, a common systemic skeletal disease, is currently treated with hormone drugs, anabolic drugs, and bisphosphonate drugs. However, long-term use of these drugs can lead to significant side effects in patients. Therefore, natural herbal preparations have been proposed as alternative treatments (Gao and Zhang, 2022).

##### 3.1.1.1 Estrogen pathway

Disordered bone metabolism is one of the causes of osteoporosis. Researchers discovered through meta-analysis that Epimedium can improve BMD and alleviate pain in patients with osteoporosis by regulating bone metabolism and significantly reducing ALP levels, resulting in satisfactory clinical efficacy (Shi et al., 2022). Decreased estrogen levels disrupt bone metabolism, a primary cause of menopausal osteoporosis (Yong et al., 2021), which Epimedium can help regulate (Li et al., 2017). Therefore, Epimedium regulates bone metabolism-related proteins, such as TRAF6, through an estrogen mechanism.

**FIGURE 2:**
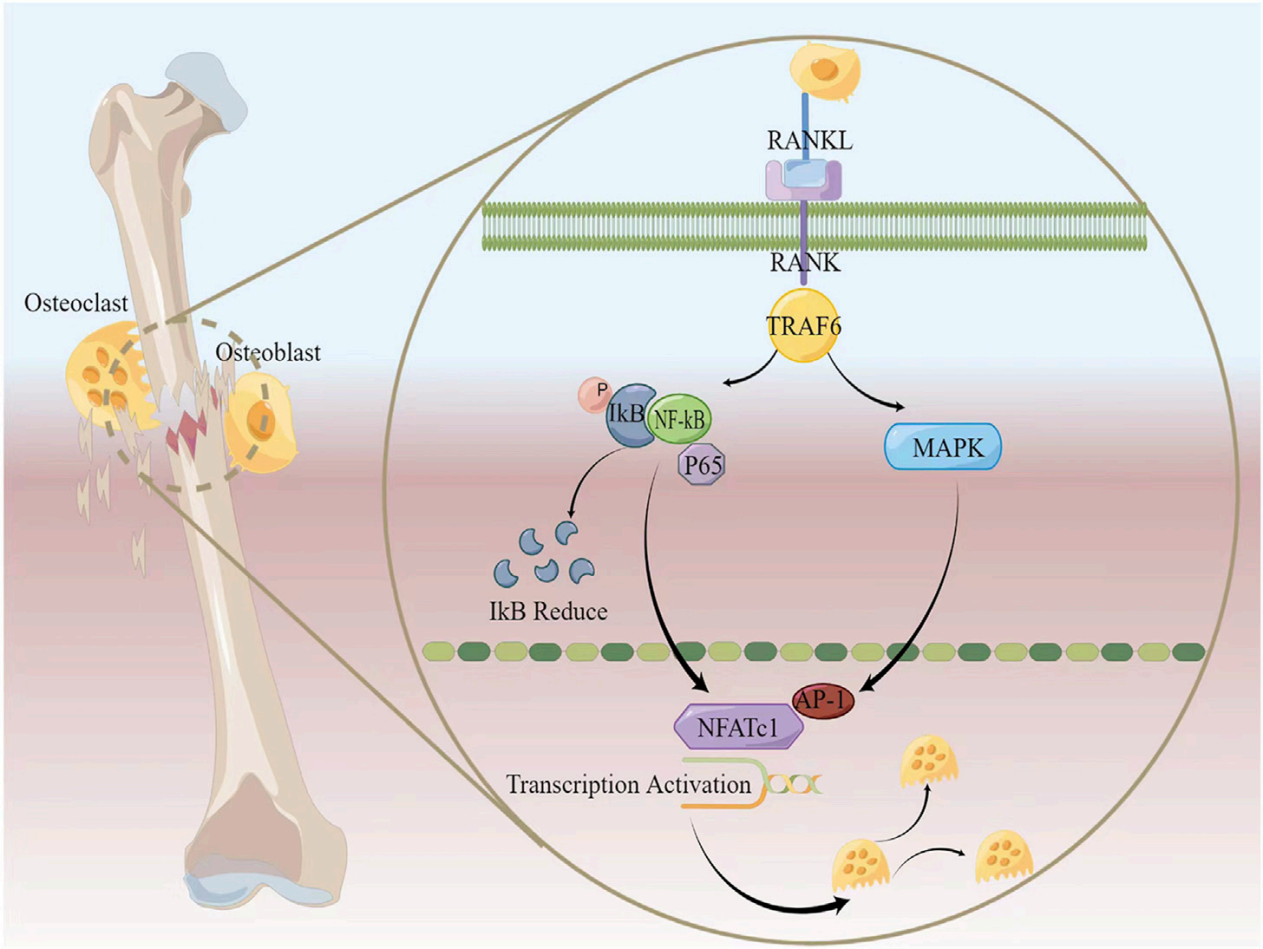
The classic mechanism of bone metabolism.

In clinical practice, the safety of icariin (ICA) in the human body has been confirmed through randomized, double-blind experiments. After taking ICA, postmenopausal female patients show increased levels of ICA metabolites detected in the serum. The bone synthesis metabolic marker BSAP level increases, while TRAF6 (a key adaptor protein that transduces RANKL/RANK signals) level decreases, indicating that ICA inhibits the improvement of osteoclast function caused by low concentrations of estrogen (Yong et al., 2021). In addition, a study indicate that icaritin (ICT) inhibited osteoclast formation in a dose-dependent manner in an OVX rat model. ICT reduced osteoclast function by downregulating TRAF6, inhibited bone resorption, prevented changes in femoral mechanical properties caused by OVX, and eliminated the increase in osteoclast formation and activity (Tan et al., 2017). NF-κB and MAPK/AP-1 signaling pathways were found to be involved in ICT restoration of OVX-induced disruption of bone metabolism (Indran et al., 2016; Tan et al., 2017). These studies suggest that Epimedium inhibits osteoclasts function by blocking TRAF6, a key protein in bone metabolism, thus exerting an anti-osteoporotic effect (Figure 2).

A study found that ICA is more effective than another flavonoid compound genistein on osteogenesis, possibly due to the isoprene group on ICA’s C-8 position, while genistein’s effect depends on its high affinity for ER receptors. However, ICA is affinity for ER is lower than that of genistein. Therefore, it is speculated that ICA is osteogenic effect also has a non-estrogenic mechanism (Ming et al., 2013). Additionally, ICT, the enzymatic hydrolysis product of ICA, has an inhibitory effect on osteoclasts formation that cannot be reversed after the use of estrogen receptor antagonist ICI182780 (Tan et al., 2017). The above results indicate that Epimedium and its extracts are not entirely transmitted through estrogen receptor signaling.

##### 3.1.1.2 Neuropeptide related mechanisms

Based on the OVX model, the serum levels of estrogen (E2), γ-carboxy-glutamic acid-containing protein (BGP), and Osteoprotegerin (OPG) in rats treated with Epimedium (0.27, 0.81, 2.43 g/kg/day) increased. The mRNA levels of related neuropeptides at the bone, brain and spinal cord were significantly elevated, upregulating spinal CGRP signaling and promoting bone growth (Liu, H. et al., 2018). In addition, neuropeptide Y (NPY) (Driessler and Baldock, 2010; Horsnell and Baldock, 2016)in the hypothalamus, calcitonin gene-related peptide (CGRP) (Liang et al., 2015), substance P (SP) (Chen et al., 2017) in the nerve fibers, and vasoactive intestinal peptide (VIP) (Mukohyama et al., 2000) in the skeletal sympathetic nerve fibers all contribute to the maintenance of bone metabolism homeostasis. This suggests that epimedium can maintain bone metabolism balance through mechanism associated with several neuropeptides involved in regulation of the brain/spinal cord/bone axis.

##### 3.1.1.3 Regulating glucocorticoid release

Secondary osteoporosis is often induced by glucocorticoids. Based on the glucocorticoid (GC)-induced osteoporosis (GIOP) model in rats, researchers found that oral administration of Epimedium and Fructus Ligustri Lucidi (100, 200 mg/kg/day) increased levels of bone formation markers such as alkaline phosphatase (ALP), bone γ-carboxyglutamic acid-containing protein (BGP), and bone mineral content (BMC), while decreasing levels of tartrate-resistant acid phosphatase (TRACP) secreted by osteoclasts. This alleviated glucocorticoid induced bone loss and bone mineral density (BMD) reduction. The preventive effect of Epimedium combined with Fructus Ligustri Lucidi on GIOP is closely related to TGF-β 1/Smads signaling pathway (Yang et al., 2017). Similarly, administration of Epimedium (10 mL/kg) to early SANFH rat models revealed increased BMD, prevention of collapse caused by osteoporosis, and inhibition of cellular autophagy by reducing the expression of related autophagy proteins, thereby exerting bone protective effects (Liu, S. et al., 2018). Additionally, studies have shown that ICA (5 × 10^−5^, 1 × 10^−4^ M) intervention can induce enhanced EVs activity and improve glucocorticoid-induced bone microvascular endothelial cells (BMECs) injury (Zhang et al., 2022).These results indicate that Epimedium can be a potential drug candidate for treating GC induced bone diseases.

##### 3.1.1.4 Hypoxic pathway

Skeletal hypoxia is also a cause of primary and secondary osteoporosis. ICA (10^−7^, 10^−6^, 10^−5^ M) can promote the differentiation of BMSCs into osteoblasts, alleviate oxidative stress and apoptosis of osteoblasts under hypoxic conditions, and preserve the osteoblasts function (Ma et al., 2014).

In a series of preclinical studies, Epimedium and its related plant metabolites have shown anti-osteoporosis effects. Although different animal models were used in these studies, the subjects were all rodents. To address this limitation, some researchers chose a non-rodent species, *Oryzias latipes*, to construct an osteoporosis model induced by Rankl. They evaluated the bone protective effect of ICA by measuring IM using specific technical procedures. The research results showed that ICA (2.5, 5, 10, 15, 20, and 25 μM) reduced the degree of mineralization matrix damage and had similar therapeutic effects to positive control drugs (25 μg/mL, Alendronate), reducing Rankl-induced bone loss (Pham et al., 2021).This indicates that Epimedium reduces bone loss in non-rodents and also has anti-osteoporosis effects.

However, the National Medical Products Administration of China released reports in 2008 and 2016 that “*Zhuanggu Joint Pill*” and “*Xianling Gubao Oral Preparation*” have caused liver damage in patients in clinical practice, and Epimedium is the main plant metabolites of these two preparations. Therefore, in the study of Epimedium’s treatment of osteoporosis, it is unclear whether researchers consider and avoid the dosage of Epimedium’s hepatotoxicity when setting animal models dosage. Researchers should study its therapeutic effects while avoiding the generation of hepatotoxic doses, which is more conducive to clinical application.

#### 3.1.2 Other bone protection

In addition to common osteoporosis, Epimedium and its extracts also have therapeutic effects on bone damage caused by other reasons. ICT, as a potential bone protectant, restores Pb-induced mineralization of bone nodules and promotes differentiation of BMECs into osteoblasts. After ICT (10 μM) treatment, Pb-induced reductions in Wnt3a and β-catenin are partially or completely, leading to the nuclear translocation of β-catenin (Sun et al., 2019). The Wnt/β-catenin is closely related to the differentiation of BMECs into osteoblasts (Fu Shuping, 2016; Oton-Gonzalez et al., 2022). Therefore, ICT can maintain bone metabolism homeostasis and alleviate Pb induced bone damage by activating the Wnt/β-catenin pathway. In addition, Epimedium flavonoids (EPF) can inhibit smoke-induced osteoclast activity by enhancing the activity of OPG and ALP, thereby inducing bone formation to prevent smoke-induced bone loss (Gao et al., 2012). ICA can reduce bone resorption induced by wear particles after total joint replacement surgery (Shao et al., 2015). These studies indicate that Epimedium has therapeutic effects on various types of bone injuries, although further experimental analysis is needed for its clinical application.

### 3.2 Reproductive system protection

#### 3.2.1 Treatment erectile dysfunction (ED)

##### 3.2.1.1 Inhibition of PDE5

ED is a common male sexual dysfunction in clinical practice, defined as the inability of the penis to achieve or maintain an erection sufficient for satisfactory sexual activity. Currently, oral administration of phosphodiesterase 5 inhibitors (PDE5Is) is the optimal method for treating ED. However, due to the side effects of PDE5Is, such as headaches, visual impairments, vomiting and diarrhea, a natural drug for the alternative treatment of ED will reduce the associated risks.

**FIGURE 3:**
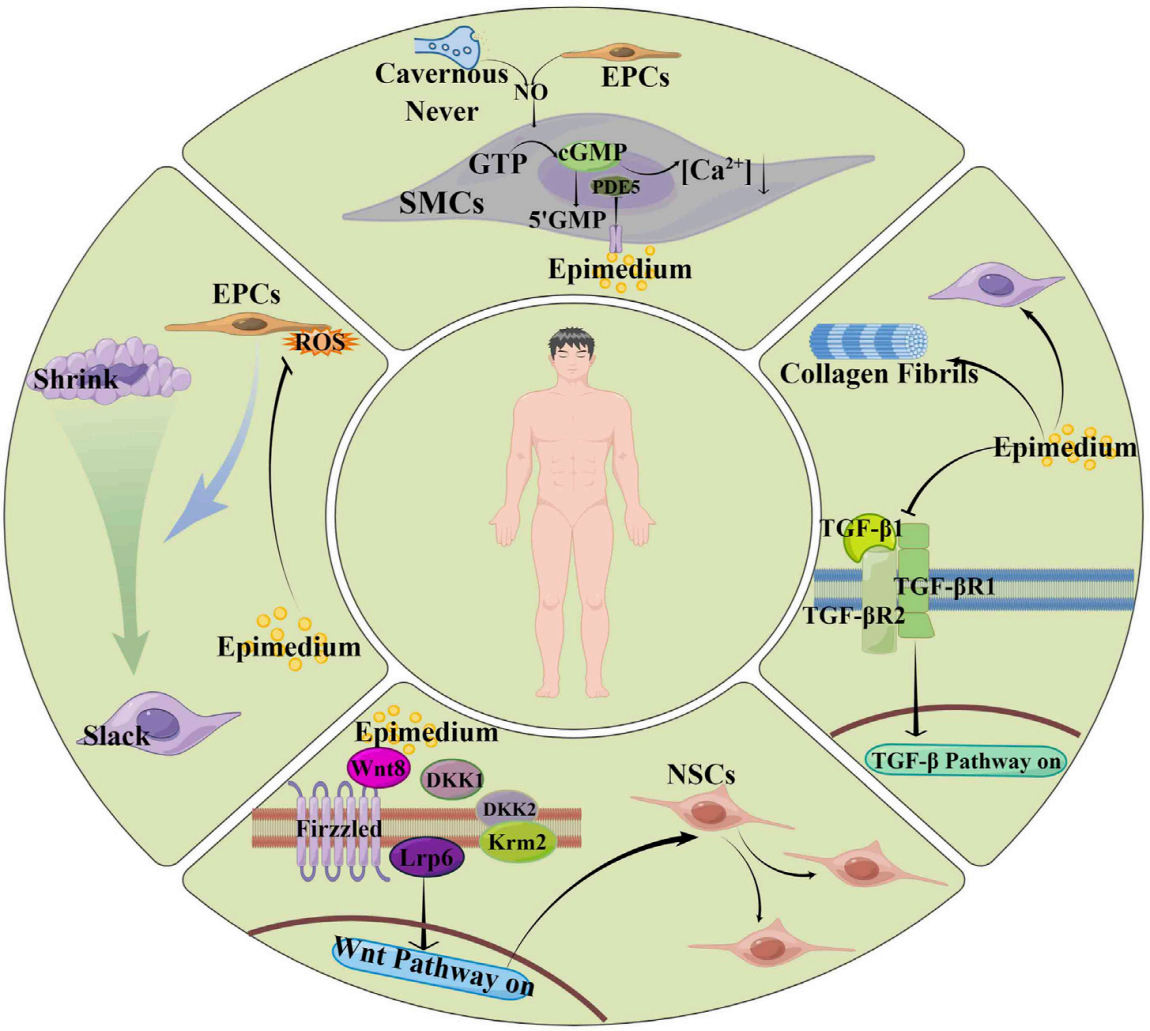
Reproductive protection mechanism of Epimedium.

Epimedium, a traditional botanical drug, is a preferred choice for tonifying the kidneys and enhancing virility (Figure 3). Modern medical research has discovered that isoflavones and biflavones in botanical drug have strong PDE5 inhibitory activity (Dell'Agli et al., 2006; Maschi et al., 2008). Epimedium contains various flavonoid components, among which EpimediumA, B, C, and rhamnosyl icariside II, ICA, and Icariside II(ICA II) are isopentenyl flavonoid glycosides (Ma et al., 2011). Therefore, Epimedium has an inhibitory effect on PDE5 *in vitro* and is a natural PDE5Is (Xin et al., 2024; Zhaojian et al., 2006) (Table 2).

**TABLE 2:** **Common dosages and models in pharmacological research on Epimedium bone and reproductive protection**.

Disease	Plant metabolites	Dosage	*In vivo*/*in vitro*	Animal models/Cells	Signaling pathway
Osteoporosis	ICT	40 mg/kg/day	*In vivo*	ovariectomized (OVX) rat model	TRAF6 NF-κB/MAPK/AP-1
Osteoporosis	water extract from Epimedium	0.27, 0.81, 2.43 g/kg/day	*In vivo*	ovariectomized (OVX) rat model	Neuropeptide
Osteoporosis	water extract from Epimedium	100, 200 mg/kg/day	*In vivo*	GIOP SD rat model	TGF-β 1/Smads
Osteoporosis	water extract from Epimedium	10 mL/kg	*In vivo*	SANFH rat model	—
Osteoporosis	ICA	5 × 10^−5^, 1 × 10^−4^ μM	*In vitro*	BMECs	—
Osteoporosis	ICA	10–7, 10–6, 10–5 μM	*In vitro*	BMSCs	Oxidative stress
Non-rodents osteoporosis	ICA	2.5, 5, 10, 15, 20 and 25 μM	*In vivo*	Medaka (*Oryzias latipes*) model	Mineralization matrix damage
Pb induced bone injury	ICT	10 μM	*In vitro*	MC3T3-E1 subclone 14 preosteoblast cell line	Wnt/β-catenin
Cigarette smoke induced bone injury	Epimedium pubescen flavonoid	75, 150, 300 mg/kg/day	*In vivo*	Passive smoking rat model	—
Osteolysis	ICA	0.1, 0.3 mg/g/day	*In vivo*	mouse calvarial model	—
ED	ICA	10^−7^,10^−6^,10^−5^,10^−4^ and 10^−3^ mol/L	*In vitro*	Rabbit Corpus Cavernosum *in Vitro*	—
ED	ICA	0.1, 1, 10 μM	*In vitro*	NSCs	Wnt and bFGF
ED	ICA/ICA Ⅱ	1, 5, and 10 mg/kg	*In vivo*	Streptozotocin-Induced Diabetic Rats model	ERK1/2
ED	ICA Ⅱ	10 mg/kg/day	*In vivo*	Type 2 diabetic rats models	—
ED	ICA	100 mg/kg	*In vivo*	DEHP induced male mice model	PI3K/Akt/eNOS Esr1/Src/Akt/Creb/Sf-1
ED	Alcohol extract from Epimedium	200, 400 mg/kg	*In vivo*	Male infertility rat model	SRIT1-HIF-1α and p53
POF	ICA	10 μg/L	*In vitro*	Rat ovary granulosa cells	secretion of estrogen and progesterone

Although the inhibitory effect of ICA on PDE5 isomers is less than half of that of sildenafil (clinically used PDE5Is), the inhibitory effect of ICA II, which is its metabolite, reaches 50% of that of sildenafil (Zhang et al., 2012). In addition, researchers have confirmed that rhamnose-based ICA II have high inhibitory activity on PDE5, and the three-dimensional quantitative structure-activity relationship (3D-QSAR) results showed that the substitution at positions C-8, C-3, C-7, and C-4 determine PDE5 inhibitory activity (Li et al., 2022). Therefore, researchers developed a novel semi-synthetic ICA analogue by modifying the C-7 and C-8 positions in the ICA structure, which has inhibitory activity similar to clinical use of PDE5Is.

Cyclic guanosine monophosphate (cGMP), a key small in the treatment of ED, can have its concentration increased by ICA. Meanwhile, PDE5 mRNA expression can also be inhibited by ICA (Zhaojian et al., 2006). In addition, flavonoids from Epimedium can activate the cGMP/PKG/Ca^2+^ signaling pathway to relax the smooth muscle of the corpus cavernosum and promote penile erection (Li et al., 2022).

In summary, Epimedium and its extracts have significant advantages in safety and efficacy as PDE5Is. In the future, Epimedium preparations may replace existing drugs for ED. However, there is limited research on how Epimedium regulates PDE5, and more experimental data is needed to clarify its regulatory mechanism.

##### 3.2.1.2 Activation of the NOS/NO/cGMP signalling pathway

During the process of penile erection, the neurotransmitter nitric oxide (NO) released by nerve endings and endothelial cells in the corpus cavernosum is crucial (Burnett et al., 1992). It not only induces the synthesis of cGMP (Chen et al., 2009), but also dilates penile blood vessels, causing arterial perfusion in the corpus cavernosum and inducing penile erection. ICA can promote the production of NO (Zhang et al., 2012) and achieve synergistic inhibition of eNOS decoupling by stimulating the production of BH4, alleviating the reduction of NO (Long et al., 2018). ICA can also control the production of NO by regulating the interaction between eNOS and proteins such as caveolin-1 and HSP90 (Liu et al., 2021). Therefore, the NO/cGMP pathway is a mechanism by which ICA promotes endogenous NO production.

##### 3.2.1.3 Improvement of pathological changes in the penis

ICA can repair damaged cavernous nerves by promoting the proliferation and differentiation of neural stem cells (NSCs). After ICA treatment, typical neural spheres appeared in the striatal single cells of naturally aborted fetuses. NSC related markers and differentiation proteins were expressed (Yang et al., 2016). Additionally, researchers have found that the novel synthesis of the ICA II configuration could treat neurogenic ED by activating the Wnt signaling pathway (Gu et al., 2021). Both studies indicate that ICA activates the Wnt and bFGF signaling pathways to induce proliferation and differentiation of NSCs and promote the generation of neurons. However, additional experimental data is needed to support the involvement of the bFGF signaling pathway.

Although ICA has been confirmed through *in vitro* experiments to have the ability to promote the proliferation and differentiation of NSCs and repair cavernous nerves, its precise mechanism of action on NSCs still needs further exploration.

The quality and quantity of endothelial cells, as well as the maintenance of the number and normal shape of smooth muscle cells (Wespes, 2002), are crucial for penile erection. The normal erection of the penis depends on the contraction and relaxation of smooth muscle cells (Dean and Lue, 2005). Studies indicate that ICA and ICA Ⅱ reverse the reduction of SMCs by regulation the TGF-β1 signaling pathway and upregulation α-smooth muscle actin (α-SMA) (Liu et al., 2011; Zhou et al., 2012). Additionally, they increase endothelial cells by down-regulating the expression of transforming growth factor β1 (TGFβ1), P-Smad2 and total Smad2 in the corpus cavernosum (Liu et al., 2011; Zhou et al., 2012). Therefore, researchers speculated that the TGF-β1 signaling pathway controls smooth muscle cell contraction by affecting EPCs. Moreover, ICA can improve endothelial dysfunction by regulating the ERK1/2 signaling pathway, inhibiting oxidative stress damage to EPCs, and promoting the proliferation and differentiation of EPCs (Tang et al., 2015). Researchers have also discovered that ICA II can reduce excessive mitochondrial autophagy in SMCs and repair SMCs damage by activating the PI3K-AKT mTOR signaling pathway (Zhang et al., 2020).

ED is a common complication in patients with diabetes (AF et al., 2010; Fedele, 2005). A series of studies have confirmed that ICA can inhibit EPC function damage caused by hyperglycemia by regulating p28/CREB, Akt/eNOS/NO, and other pathways. Therefore, it is speculated that ICA and ICAⅡ have great potential in treatment of ED, and further research is needed in the future.

Fibrosis of the corpus cavernosum is one of the main causes of ED. Research has demonstrate that ICA II can increase the ratio of smooth muscle cells to collagen fibers, thereby improving ED (Zhang et al., 2020). However, the mechanism by which ICA II inhibits corpus cavernosum fibrosis is still under research.

In the future, the high expression of TGF-β1 and connective tissue growth factor (CTGF) (Moreland, 1998; Qabazard et al., 2021) in ED rat models and LOX activation leading to penile fibrosis (Wan et al., 2018) maybe potential mechanisms through which ICA inhibits corpus cavernosum fibrosis.

##### 3.2.1.4 Promotion of testosterone synthesis

The decrease in testosterone is an important factor of ED. Testosterone, a type of steroid hormone, is usually secreted by Leydig cells in testis. Research has discovered that Epimedium extract enhances the secretion of testosterone in Leydig cells (Sun et al., 2022). ICA can promotes testosterone production by activating the Esr1/Src/Akt/Creb/Sf-1 signaling pathway (Sun et al., 2019; Sun et al., 2022). In addition, studies have indicate that NOS/NO/cGMP may be an autocrine pathway for testicular steroids, suggesting that ICA can also promote testosterone secretion through this signaling pathway (Andric et al., 2010).

##### 3.2.1.5 Improving sperm development and repairing testicular damage

Oxidative stress is an important cause of male infertility (Smits et al., 2022), therefore, antioxidant therapy is commonly used in clinical practice. Studies have shown that Epimedium extract restores male rat sperm cell apoptosis caused by LHRH through increasing SOD level and decreasing 8-OHDG (reactive oxygen species) levels (Park et al., 2017; Zhao et al., 2017). By exerting antioxidant capacity, Epimedium extract can also reverse oxidative stress-induced testicular tissue atrophy and decrease SOD and CAT activities in rats (Khan and Ahmed, 2009; Munir et al., 2020), inhibit P16-CDK6 expression, testicular ROS activity, and DNA oxidative damage in spermatogenic cells, and improve sperm deformity (Zhao et al., 2022). Additionally, researchers have also discovered other mechanisms by which Epimedium extract reduces oxidative damage to testicular DNA in aging rats, such as inhibiting the endogenous mitochondrial apoptosis pathway in the testis by activating SRIT1-HIF-1α (He et al., 2021) and suppressing the p53-dependent pathway that regulates DNA oxidative damage (Gambino et al., 2013; Speidel, 2015; Zhao et al., 2017).

Sertoli cells protect sperm development and provide nutrition (MD., 1998). Research has shown that ICA and ICA II can restore the reduction in Sertoli cells caused by streptozotocin and regenerate sperm activity (He et al., 2021; Xu et al., 2014). ICA can improve sperm quality and quantity by activating the ERK1/2 signaling pathway (Nan et al., 2014).

#### 3.2.2 Female reproductive system protection

Premature ovarian failure (POF) refers to ovarian dysfunction. Women with POF typically exhibit ovarian infertility and low fertility (Cong et al., 2020). Epimedium can exert its bone-protective ability through the estrogen pathway. Therefore, researchers further studied the effects of Epimedium on the ovaries by promoting estrogen production. The results showed that Epimedium upregulates the expression of CYP17 and CYP19 at the mRNA and protein level, which control estrogen biosynthesis, promotes the proliferation of oocyte granules, and the secretion of estrogen and progesterone, thereby improving ovarian endocrine function (Nie et al., 2019).

In clinical practice, thinning of the endometrium is an important factor leading to female infertility (Bu et al., 2019). Research has demonstrate that the expression of PI3K, AKT, and p-AKT proteins in thin endometrium is lower than that in normal endometrium (Le et al., 2016). Previous studies have revealed that ICA can activate the MEK/ERK and PI3K/Akt/eNOS signaling pathways that regulate angiogenesis (Chung et al., 2008). Therefore, researchers speculated that ICA can activate this signaling pathway to promote endometrial angiogenesis and increase endometrial thickness. The test results of thin endometrial cells treated with ICA confirmed this hypothesis (Le et al., 2015; Le et al., 2017).

Although the above studies have demonstrated the protective effect of Epimedium on the female reproductive system, more aspects need to be thoroughly investigated: Are there other means by which Epimedium increase the thickness of the endometrium aside from promoting blood vessels formation? What are the mechanisms and related targets of Epimedium in improving POF? Additionally, as discussed above, Epimedium improves sperm development and repairs pathological changes in the corpus cavernosum through antioxidation. Similarly, dose Epimedium have therapeutic effects on female egg development and tubal damage, and what are the specific mechanism? These questions need to be further explored.

### 3.3 Anti-tumour activity

#### 3.3.1 Acts on tumour cell proliferation and apoptosis

Apoptosis is a type of programmed cell death that is self-ordered and controlled by genes. In recent years, many studies have shown that the plant metabolites of Epimedium can induce tumor cell apoptosis and inhibit tumor proliferation by regulating multiple signaling pathways.

##### 3.3.1.1 PI3/AKT/mTOR signal pathway

Network pharmacology enrichment analysis revealed that the main active components of Epimedium are primarily associated with proteins in the PI3K/AKT signaling pathway. Therefore, *in vitro* experiments were conducted on this pathway, revealing that ICA can make SKOV3 cell nuclei dense and fragmented, inhibit cell proliferation, migration, and invasion, increase cell apoptosis rate by regulate the PI3K/AKT signaling pathway (Wang, S. et al., 2020). In addition, ICA can regulate the apoptosis and proliferation of OC cells by regulating microRNA-21 and its target genes PTEN, RECK, and Bcl-2 (Li et al., 2015). Therefore, the PTEN/PI3K/AKT pathway may be a key signaling pathway for the treatment of OC with Epimedium. In another study, ICAⅡ activates caspase-dependent apoptosis through the mTOR apoptotic signaling pathway and inhibits HCC proliferation by the inhibiting the NF- кB signaling pathway (Guo et al., 2020; Sun et al., 2021).

Furthermore, researchers have found that ICT induces apoptosis in cisplatin resistant OC cells by activating the p53 apoptotic pathway while inhibiting the Akt/mTOR signaling pathway (Gao et al., 2018), indicating that ICT has the potential to address the resistance limitations of the chemotherapy drug cisplatin in clinical use.

##### 3.3.1.2 MAPK/ERK signal pathway

In a series of studies based on breast cancer cells, ICT reduced the expression of ER- α36, a key protein responsible for the development of triple-negative breast cancer (TNBC), as well as the expression of epidermal growth factor receptor (EGFR). It also inhibited the ER-α36-mediated induction of the MAPK/ERK pathway and Cyclin D1, thereby inhibiting cancer cell proliferation and promoting its apoptosis (Wang et al., 2017). In addition, ICT can enhance the radiosensitivity of cancer cells and has a synergistic effect with ionizing radiation (IR), which is currently a promary treatment for malignant tumors. ICT inhibits tumor proliferation by inhibiting the activation of ERK1/2 and AKT pathways induced by IR and blocking the G2/M phase of cancer cells (Hong et al., 2013).

##### 3.3.1.3 ROS mediated signaling pathways

The increase of ROS is a prominent feature of various types of cancer (Moloney and Cotter, 2018). Research has demonstrate that HCC, after long-term ICT treatment, shows significant ROS accumulation, increased formation of γH2AX fluorescent dots, a large number of cells aggregation in the G0/G1 phase, and a decrease in S and G2/M phase cells (Wang et al., 2019). Therefore, ICT can induce ROS production and cell cycle arrest, causing DNA damage and aging in HCC.

In the treatment of cervical cancer cells (CCA), ICT activates the mitochondrial/Caspase apoptosis pathway and inhibits CCA proliferation by activating the AKT/Cyclin E/CDK2 signaling pathway (Chen et al., 2019; Sun et al., 2020b). However, in lung cancer cells, in addition to ICA induced apoptosis through the MAPK/ERK pathway, as previously mentioned (Zheng et al., 2014), researchers have discovered that the apoptotic effect of ICAⅡ on cancer cells depends on the activation of JNK and p38MAPK downstream of ROS. When using inhibitors of p38MAPK and JNK, the apoptotic effect of ICAⅡ on cancer cells almost disappears (Song et al., 2012). Therefore, the ROS/MAPK/JNK signaling pathway is another mechanism by which Epimedium treats lung cancer.

#### 3.3.2 Inhibition of tumour cell metastasis

A certain concentration of ICA II can inhibit tumor cell migration by regulating the MMP2/9 signaling pathway through JNK (Sun et al., 2020a). Additionally, Epimedium extract and ICA II can regulate tumor development, survival, and metastasis through inhibiting the NF-κB signaling pathway (Kim and Park, 2014; Lee et al., 2017; Shi et al., 2020).

Epithelial mesenchymal transition (EMT) is one of the markers of cancer metastasis (Talmadge and Fidler, 2010). ICT inhibits the invasion of Glioblastoma multiforme (GBM) cells by downregulating the expression of EMMPRIN. Further analysis reveals that ICT regulates EMMPRIN (matrix metalloproteinase inducer)) through the PTEN/Akt/HIF-1α pathway (Xu et al., 2015).

Additionally, ICT promotes HCC apoptosis through the p53/alpha-fetoprotein (AFP) pathway and indirectly affects protein expression related to tumor development, apoptosis, and migration by regulating HMG-box transcription factor 1 (HBP1) and AFP (Cao et al., 2021; Li et al., 2021).

In summary, Epimedium and its extracts can induce cancer cell apoptosis and inhibit cancer cell proliferation in different types of tumors through different signaling pathways, generally involving the mitochondrial/Caspase, MAPK/ERK, PI3K/AKT, ROS signaling pathways (Table 3).

**TABLE 3:** Partial monomeric plant metabolites and related signaling pathways of *Epimedium Folium* in exerting anti-tumor effects.

Chemical structure	Disease	Plant metabolites	Signaling pathway
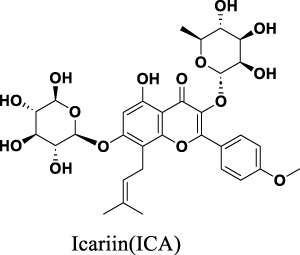	liver cancer	ICT	Caspase-8
			P53/AFP
			Fas
			ROS
		ICA II (Baohuoside I)	mTOP
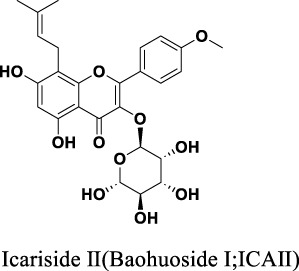			NF-κB
	Breeast Cancer	ICT	MAPK/ERK
			AKT
	OC	ICA	PI3/AKT
	—	ICT	p53
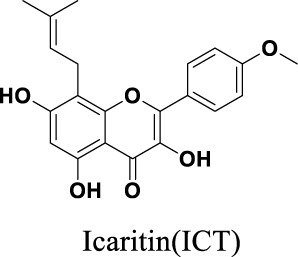			AKT/mTOR
	—	ICA II (Baohuoside I)	MMP2/9
	—	ICA II (Baohuoside I)	NF-κB
	—	ICT	PTEN/AKT/HIF-α
			ERK
			IL-6/Jak2/Stat3

#### 3.3.3 Other mechanisms

Inhibiting cancer stem cells (CSCs) and immune regulation are new perspectives for treating cancer. CSCs are tumor cells with stem cell characteristics that can promote cancer cell resistance, metastasis, and recurrence in cancer treatment (Najafi et al., 2019). Studies indicate that ICT not only induces apoptosis of non-breast cancer stem cells (BCSCs) through the ERK pathway, but also inhibits the growth of breast cancer stem cells with high ALDH (aldehyde dehydrogenase, BCSCs marker) (Guo et al., 2011). For hepatocellular carcinoma initiating cells (HCICs), ICT inhibits the malignant proliferation of HCICs through the IL-6/Jak2/Stat3 pathway, reducing the proportion of EpCAM (HCIC marker) positive cells (Zhao et al., 2015). Both studies suggest that Epimedium and its extracts have great potential in treating cancer by inhibiting CSCs. However, there is currently limited data and all findings come from *in vitro* experiments. Therefore, extensive *in vivo* and *in vitro* experiments are needed for further evaluation.

During cancer development, tumor cells evade the host immune system to maintain growth, leading to the application of tumor immunology in cancer treatment. It is discovered that Epimedium has a regulatory effect on immune function. ICA can increase the number of CD3^+^/CD69^+^and CD69^+^/NKG2D^+^ lymphocytes in mouse spleen lymphocytes, as well as the activity of cytotoxic lymphocyte (CTL). The host’s immune function against cancer is enhanced and the immune escape of cancer cells is suppressed after treatment with ICA (Zhang et al., 2018). ICT can induce anti-tumor immunity by inhibiting extramedullary hematopoiesis (EMH) in the spleen, thereby inhibiting myeloid derived suppressor cells (MDSCs) that protect cancer cell immune escape (Tao et al., 2021). In recent years, studies have found that ICA II and ICA I can trigger immune mediated IDILI, which enhances the immune function of the body by activating the NLRP3 inflammasome, leading to liver injury (Gao et al., 2021; Wang Z. et al., 2020). Therefore, in clinical practice, the application of Epimedium has gradually become a double-edged sword, and researchers need to pay attention to its toxic effects when applying Epimedium and its related preparations in different neighborhoods. The above indirect explanation, ICA and ICT can be used in the cancer treatment by modulating immune functions.

Additionally, the mechanism of Epimedium inducing apoptosis and inhibiting the proliferation of bladder cancer differs from other types of cancer. Studies confirm that ICT reduces the ATP that maintains tumor cell growth by reducing the MMP of cancer cells, thereby inhibiting the proliferation of bladder cancer cells. This mechanism does not involve ROS/JNK and other related signaling pathways (Pan et al., 2016).

In summary, the targets and potential mechanisms of the anti-tumor effect of Epimedium are diverse, and the same components have specificity in targeting different types of tumors. For example, ICT can treat OC by regulating the p53/AKT/mTOR signaling pathway (Gao et al., 2018), inhibit breast cancer cell proliferation through MAPK/ERK signaling pathway (Wang et al., 2017), and treat HCC by inducing ROS production and cell cycle arrest (Wang et al., 2019). ICT can also act on the host’s immune function to achieve tumor cure (Tao et al., 2021).

However, current research on the anti-tumor effect of Epimedium is mostly based on *in vitro* experiments, which have significant differences from the mode of action of drugs *in vivo*, and most experimental designs lack the setting of a positive control group. Therefore, relevant *in vivo* experiments are needed to verify the conclusions drawn from *in vitro* experiments and supplement the experimental groups to obtain more reliable experimental conclusions.

### 3.4 Anti-inflammatory

The Inflammatory response is a manifestation of the normal physiological defense mechanism in human body. When there are excessive inflammatory mediators, immune and inflammatory diseases occur. The active metabolites of Epimedium have anti-inflammatory effects to varying degrees. Epimedium water extract inhibits the inflammatory mediators NO, IL-6, and IL-1 induced by lipopolysaccharide (LPS) in macrophages by inhibiting the NF-κB/MAPK pathway. In addition, Epimedium water extract reduces xylene induced ear edema in mice (Oh et al., 2015). ICA inhibits NF-κ B/MAPK pathway by interfering with ERK and p38 phosphorylation and reduces the production of inflammatory factors by promoting the nuclear translocation of glucocorticoid receptors α (GRα) and increased GRα in the nucleus bound more NF-κB, c-Jun, and Stat3, promoted glucocorticoid receptor (GR)function, thereby exerting anti-inflammatory activity (Sun et al., 2018). In addition, ICA can reduce the production of TNF-α and IL-8 in cigarette smoke (CS) induced mouse serum and A549 cells, inhibit NF-κB p65 protein phosphorylation and block IκB-α protein degradation, and alleviate CS induced lung inflammation. ICA also restored the expression of GR protein and mRNA, which will help alleviate inflammation (Li et al., 2014). Furthermore, ICA can also improve CYP-induced acute cystitis and LPS-induced endometritis and peritonitis in mice (Amanat et al., 2022; Huang et al., 2018; Shaukat et al., 2022).

In neurological diseases, total flavonoids of Epimedium exhibit beneficial biological activities against central nervous system demyelinating diseases, inhibiting neuroinflammatory responses to protect the myelin sheath (Meng-Ru et al., 2021). ICA II exerts neuroprotective effects during cerebral ischemia/reperfusion by inhibiting inflammation and inducing cell apoptosis (Deng et al., 2016). Therefore, an investigation into cognitive impairment in Alzheimer’s disease indicate that the reversal of β-Amyloid protein-induced cognitive impairment by ICA II is also achieved by inhibiting neuroinflammation, repairing nerve damage, and promoting cell apoptosis (Deng et al., 2017). Further research on the anti-inflammatory activity of Epimedium has discovered that Epimedium can alleviate inflammatory reactions in diseases such as neurological disorders, osteoarthritis, and various cancers. Epimedium has high value in anti-inflammatory applications.

### 3.5 Antiviral

Research has demonstrated that epimedium water extract has an inhibitory effect on influenza A. It inhibits influenza virus protein expression in a dose-dependent manner, suppresses cellular lesions and cytopathy caused by H1N1, H3N2, and Influenza B virus. In addition, at a dose of epimedium water extract of 100 μg/mL, the expression inhibition rate of influenza virus is 90%, reduces the hemagglutinin (HA) and neuraminidase (NA) of H1N1 influenza virus, and inhibits virus adhesion to cells (Cho and Ma, 2022). In addition, Epimedium exhibits broad-spectrum antiviral activity both *in vivo* and *in vitro*. It significantly reduces the replication of influenza A virus PR/8, vesicular stomatitis virus (VSV), herpes simplex virus (HSV) and Newcastle disease virus (NDV) in cells by promotes phosphorylation of IRF-3, STAT1, and TBK1, activating the type I IFN signaling pathway, inducing the release of type I interferon (IFN-I) and pro-inflammatory factors. Mice orally administered Epimedium can combat influenza A subtypes such as H1N1, H5N2, H7N3, and H9N2 (Cho et al., 2015). In 2012, the chicken embryo fibroblast monolayer was co administered with Epimedium Flavones, Newcastle disease virus (NDV), and ND vaccine. Cellular A570 values were measured and it was found that Epimedium Flavones significantly increased cellular A570 values, inhibit the cellular infectivity of NDV,improve the protective effect of ND vaccine (Yuqing et al., 2012). These studies suggest that Epimedium has antiviral properties and can be developed as a natural virus inhibitor.

## 4 Toxicity

Epimedium is a traditional “non-toxic” traditional Chinese medicine commonly used in clinical practice to treat diseases such as osteoporosis and ED. In recent years, the clinical preparations of Epimedium such as “*Zhuanggu Joint Pill*” and “*Xianling Gubao Oral Preparation*” have been issued adverse reaction notices by the National Medical Products Administration of China. Among them, 2665 cases showed that “*Xianling Gubao Oral Preparation*” damaged the liver, gallbladder, and gastrointestinal systems in patient. Therefore, the safety assessment of Epimedium and the confirmation of potential toxic components have been overlooked.

In 2019, *In vitro* experiments have found that the flavonoid compound ICA Ⅱ of Epimedium exhibits significant cytotoxicity (Zhang et al., 2019). In 2023, Researchers conducted a 13 weeks subchronic toxicity experiment using SD rats with Epimedium water extract (7.5, 15, or 30 g/kg). The results showed that after 13 weeks of administration, the no-observed-adverse-effect level was not determine but caused liver and adrenalgland damage in SD rats (Song et al., 2023). Zebrafish embryos were exposure to flavonoid compound ICA (0, 2.5, 10, and 40 μM) of Epimedium, reduced hatching rates and disrupted thyroid endocrine function after treatment with 10 μM and 40 μM ICA, indicating that ICA disrupts thyroid development and hormone synthesis, leading to developmental toxicity (Wu et al., 2023). In addition, in the study of the detoxification mechanism of the combination of *Epimedium and Ligustri lucidi fructus*, *Ligustri lucidi fructus* could significantly reduce the concentration of flavonoid compound ICA, epimedin A, epimedin B, epimedin C, ICAⅡ of Epimedium in rats, attenuate their virulence (Wang et al., 2022).

In short, Epimedium is toxic, and its flavonoids may also be potential toxic components. However, the contraindications for the use of Epimedium are unclear, and the clinical safe dosage is also unknown. In traditional model organisms, its toxicity may not be obvious, but in new model organisms such as zebrafish, organoids, Organs-on-chips (OoCs), etc., it may be more sensitive to its toxicity. Therefore, further evaluation is needed to confirm the toxic components and dose safety of Epimedium.

## 5 Discussion

Chinese medicine considers the kidney to stores essence, which generates marrow, leading to bone synthesis, Therefore, the reproductive protection, bone protection, and anti-tumor pharmacological effects of Epimedium are all research hotspots. This article summarizes the plant metabolites, model, and possible experimental design limitations of the article by reading and organizing relevant literature (Table 4). In bone protection, the anti-osteoporosis effect of Epimedium has been discussed in detail from the perspective of bone metabolism. Epimedium can inhibit the function of osteoclasts by regulating estrogen levels, maintain the function of osteoblasts through antioxidant stress, and achieve bone protection, inhibition of bone loss, and bone autophagy. In addition, Epimedium has non estrogenic pathways and related neuropeptide mechanisms for bone metabolism balance, and the active metabolites of Epimedium have shown significant bone protection effects in both vertebrates and invertebrates. There are already drug formulations of Epimedium for treating osteoporosis in the market, such as “*Xianling Gubao Oral Preparation*” and “*Zhuanggu Joint Pill*”. However, long-term use has been found in clinical practice to cause liver damage in patients. Therefore, *in vivo* studies on the bone protective effect of Epimedium need to be conducted under the premise of safe dosage, and further clarification is needed on the toxic components in Epimedium and whether the toxicity of Epimedium can be reduced through interaction with traditional Chinese medicine. In the future, experiments on the bone protective effect of Epimedium may be able to detect relevant liver injury indicators to improve possible doubts in experimental design. In the reproductive process, sildenafil, tadalafil, and vardenafil are all PDE5Is drugs commonly used in clinical practice to treat ED. However, these drugs can cause side effects such as headaches, visual impairment, vomiting, and diarrhea. Therefore, seeking natural PDE5Is as a substitute for treatment will greatly reduce drug risks. Research has found that, the active metabolites of Epimedium improve reproductive system damage in both men and women through different mechanisms. Epimedium can improve the pathological changes of the penis, promote testosterone synthesis, promote sperm development, repair testicular damage, and enhance the secretion of estrogen and progesterone to improve ovarian endocrine function. It is a candidate drug for the treatment of ED and female reproductive diseases. However, research on the protection of the female reproductive system deserves more attention. Additionally, epimedium related preparations have produced toxic side effects in the clinical treatment of bone diseases, and the toxic components are unclear. Therefore, in terms of reproductive effects, it should also be suspected whether there are potential toxic effects or whether the toxicity of Epimedium related preparations may be caused by other traditional Chinese medicine components in the preparation or the repulsion between traditional Chinese medicines. These doubts need further exploration and clarification by researchers. Although there is controversy over the toxic side effects of Epimedium, there is sufficient research data for treating ED and osteoporosis of Epimedium, and the plant metabolites are clear, which may accelerate the development of new drugs to treat these diseases in further clinical studies.

**TABLE 4:** Experimental models and limitations in some papers.

Plant metabolites	Model	Limit	Reference
ICA	Mouse Leydig cell	It is unclear whether ICA regulates Leydig cells through the cAMP-PKA signaling pathway and inhibits ROS effects	Sun, J. et al. (2019)
	ICR male		
ICAⅡ	SD rats	The research on the mechanism of action is not thorough enough, and the efficacy and therapeutic targets need to be further clarified	Gu et al. (2021)
ICA	Male SHR rats	Further research is needed on the protein-protein interactions related to the mechanism of action	Liu et al. (2021)
ICA	Male SHR rats	Lack of *in vitro* research	Long et al. (2018)
Hydroethanolic extract of Epimedium	Male albino rats	Lack of research on some mechanisms	Munir et al. (2020)
ICA	SD rats	Lack of research on some mechanisms	He et al. (2021)
Epimedium granules	peroxisomal biogenesis factor 3 (Pex3)-knockout (KO, −/−) mice	It is necessary to supplement the administration of Epimedium in mice of different ages to further verify whether the mechanism involved is age dependent	Zhao et al. (2022)
ICAⅡ	Male Wistar rats	—	Zhang et al. (2020)
Epimedium extracting solution	SD rats	Lack of research on upstream and downstream proteins related to the induction of autophagic protein apoptosis in Epimedium	Liu, S. et al. (2018)
ICA	Medaka fish (*Oryzias latipes*)	—	Pham et al. (2021)
ICT	MC3T3-E1 subclone 14preosteoblast cell	The activation of the Wnt pathway is unclear	Sun, K. et al. (2019)
ICA	BMSCs	—	Shuping et al. (2016)
ICAII	Female BALB/c nude mice	Additional knockout experiments on MMP2/9 can be conducted to further confirm the relevant conclusions	Sun et al. (2020a)
ICT	HepG2 cell and SMMC7721	—	Li et al. (2021)

In anti-tumor research, the active metabolites of Epimedium target different pathways. Among the apoptosis-inducing pathways, ERK, JNK, and p38 MAPK of the MAPK subfamily are the most studied and most widely involved pathways. Epimedium Folium induces not only endogenous apoptosis (mitochondria/Caspase) but also exogenous apoptosis in cancer cells. In addition to the standard cancer therapeutic pathways, the active metabolites of Epimedium also inhibit cancer cells through immunomodulation and inhibition of the CSCs, Therefore, as a commonly used tonifying traditional Chinese medicine in clinical practice, whether Epimedium can achieve tumor treatment by enhancing the body’s immune function and causing immune dysfunction still requires experimental data support. In addition, some monomeric components in Epimedium have a risk of causing liver damage, and further research is needed to determine whether Epimedium can cure tumors through its toxic effects. For its anti-inflammatory activity, Epimedium can alleviate central nervous system demyelinating diseases and Alzheimer’s disease, covering a wide range of conditions. Regarding antiviral activity, the composition of Epimedium extract is unclear, and data is scarce, therefore, further research on the active substances is suggested.

## 6 Conclusion

Epimedium, as a traditional tonifying traditional Chinese medicine, its extract and related compounds exhibit various biological functions, such as anti osteoporosis, improving ED, anti-tumor, anti-inflammatory, etc. The flavonoids in Epimedium are the main force in exerting various pharmacological effects, therefore, Epimedium flavonoids are expected to be developed into clinical preparations for treating various diseases.

In recent years, Epimedium has been clinically applied in the reproductive system and bone protection, but due to its potential toxic effects, further research is needed on its toxicological characteristics. In addition, Epimedium has shown great potential in anti-tumor effects, but so far, most studies have been conducted *in vitro*. Therefore, *in vivo* experiments are needed to verify its anti-tumor effects. There are relatively few *in vivo* and *in vitro* experimental studies on the anti-inflammatory activity of Epimedium, and research on its anti-inflammatory activity may be conducted under pathological conditions.

This article discusses the main pharmacological effects and related mechanisms of Epimedium in the last decade, providing a reference for subsequent pharmacological effects and mechanism research of Epimedium.

## Figures and Tables

**Table:** 

**ALP**	Alkaline phosphatase
**AFP**	Alpha-fetoprotein
**BGP**	Bone γ-carboxyglutamic acid-containing protein
**BMC**	Bone mineral content
**BMD**	Bone mineral density
**BMECs**	Bone microvascular endothelial cells
**BCSCs**	Breast cancer stem cells
**CGRP**	Calcitonin gene related peptide
**CGMP**	Cyclic guanosine monophosphate
**CTGF**	Connective tissue growth factor
**CCA**	Cervical cancer cells
**CSCs**	Cancer stem cells
**CTL**	Cytotoxic lymphocyte
**EPF**	Epimedium Folium flavonoids
**ED**	Erectile dysfunction
**EPCs**	Endothelial progenitor cells
**EGFR**	Epidermal growth factor receptor
**EMT**	Epithelial mesenchymal transition
**EMH**	Extramedullary hematopoiesis
**GIOP**	Glucocorticoid-induced osteoporosis
**GC**	Glucocorticoid
**GBM**	Glioblastoma multiforme
**GRα**	Glucocorticoid receptors α
**HCC**	Hepatocellular carcinoma
**HBP1**	HMG-box transcription factor 1
**HCICs**	Hepatocellular carcinoma initiating cells
**HA**	Hemagglutinin
**HSV**	Herpes simplex virus
**ICA**	Icariin
**ICT**	Icaritin
**ICA II**	Icariside II
**IR**	Ionizing radiation
**LPS**	Lipopolysaccharide
**MDSCs**	Myeloid derived suppressor cells
**NPY**	Neuropeptide Y
**NO**	Nitric oxide
**NSCs**	Neural stem cells
**NA**	Neuraminidase
**NDV**	Newcastle disease virus
**OPG**	Osteoprotegerin
**PDE5Is**	Phosphodiesterase 5 inhibitor
**POF**	Premature ovarian failure
**ROS**	Reactive oxygen species
**SP**	Substance P
**TRACP**	Tartrate-resistant acid phosphatase
**TGFβ1**	Transforming growth factor β1
**TNBC**	Triple-negative breast cancer
**IDILI**	Idiosyncratic drug induced liver injury
**VIP**	Vasoactive intestinal peptide
**VSV**	Vesicular stomatitis virus
**3D-QSAR**	Three-dimensional quantitative structure-activity relationship
**α- SMA**	α- Smooth muscle actin
**DEHP**	Di (2-ethylhexyl) phthalate
